# Whole exome analysis reveals the genomic profiling related to chemo‐resistance in Chinese population with limited‐disease small cell lung cancer

**DOI:** 10.1002/cam4.4950

**Published:** 2022-06-23

**Authors:** Jiangyong Yu, Shuangtao Zhao, Zhe Su, Chengli Song, Lihong Wu, Jingbo Wang, Nan Bi, Lvhua Wang

**Affiliations:** ^1^ Department of Medical Oncology, Beijing Hospital, National Center of Gerontology, Institute of Geriatric Medicine Chinese Academy of Medical Sciences Beijing China; ^2^ Department of Thoracic Surgery, Beijing Tuberculosis and Thoracic Tumor Research Institute/Beijing Chest Hospital Capital Medical University Beijing China; ^3^ Peking‐Tsinghua Center for Life Science, Academy for Advanced Interdisciplinary Studies Peking University Beijing China; ^4^ Novogene Bioinformatics Institute Beijing China; ^5^ Burning Rock Biotech Guangzhou China; ^6^ Department of Radiation Therapy, Cancer Hospital, Chinese Academy of Medical Sciences Peking Union Medical College Beijing China

**Keywords:** chemotherapy resistance, neo‐adjuvant therapy, small cell lung cancer, whole exome sequencing

## Abstract

**Purpose:**

The mechanism of chemo‐resistance in small cell lung cancer (SCLC) is unclear. This study aims to explore the resistance‐related genomic profiles of residual tumors after neo‐adjuvant chemotherapy (NAC) in SCLC through the whole‐exome sequencing (WES).

**Experimental design:**

A total of 416 limited diseases (LD) SCLC patients underwent surgery were retrospectively analyzed, of which 40 patients received NAC. Then we selected 29 patients undergoing NAC (*n* = 19) and chemotherapy naïve (CTN, *n* = 10) to perform WES sequence with formalin‐fixed paraffin‐embedded samples including tumor and paired para‐tumor.

**Results:**

In total, single nucleotide variation and mutation rate were similar between NAC and CTN groups. The mutation signatures were significantly discrepant between NAC and CTN groups, as well as among patients with partial response (PR), stable disease (SD), and progressive disease. There were more copy number variation deletions in NAC group compared with CTN group. The inactivation of *TP53* and *RB1* were the most significantly events in both NAC and CTN groups. *RB1* nonsense mutations were recurrent in NAC group (9/19 vs. 0/9, 47.4% vs. 0%) with favorable survival, while the frame‐shift deletions were frequent in CTN group (3/9 vs. 3/19, 33.3% vs.15.8%). Integrated function enrichment revealed that the frequently mutant genes were involved in cell cycle, metabolic reprogramming, and oncogenic signaling pathways in NAC group, such as BTG2 pathway, glycolysis in senescence and P53 pathway. A total of 27 genes presented frequently mutant in NAC group and might played a positive role in drug resistance. Multiple genes including *BRINP3*, *MYH6*, *ST18*, and *PCHD15*, which were associated with prognosis, occurred mutant frequently in PR and SD groups.

**Conclusion:**

Residual tumors after neo‐adjuvant therapy exhibited different mutation signature spectrum. Multiple genes including *RB1* nonsense mutations, *BRINP3*, *MYH6*, *ST18*, and *PCHD15* were with frequent mutation in residual tumors, which might participate chemo‐resistance and influenced the prognosis in patients with limited disease SCLC.

## Graphical abstract

Genomic alterations related to chemo‐resistance in small cell lung cancer.

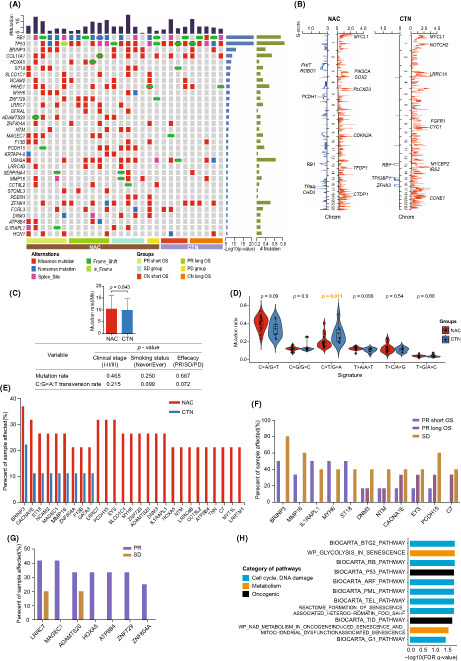

## 1 INTRODUCTION

Small‐cell lung cancer (SCLC) accounts for 13%–15% of all new lung cancer patients with an incidence of more than 180,000 patients each year.^1^ As a malignant tumor with early metastasis and rapid development, little progress in SCLC treatment was acquired in the past three decades.^2^ Immune checkpoint inhibitor (ICIs) altered the treatment paradigm of many tumors. However, atezolizumab‐based combination recommended as first‐line treatment in SCLC only brought overall survival (OS) 2 months longer compared with chemotherapy alone.^3^ Chemotherapy is still the most important treatment of SCLC.

Although initial treatment with platinum‐coupled chemotherapy has a significant response, few patients with SCLC achieve a complete response.^4^, ^5^, ^6^ More than 85% SCLC patients relapse within 2 years and develop resistance to further treatment, resulting in a response of less than 20% and a worse prognosis.^7^ Furthermore, exploration of the mechanisms associated with drug resistance and relapse is the key to overcome the dilemma of SCLC treatment. However, the lack of surgical tissue, especially residual and recurrent tumors, severely restricts the study on the mechanism of drug resistance in SCLC. The genomic pattern of relapsed SCLC and the molecular mechanisms driving chemotherapeutic resistance remain unclear.

In the past decade, few studies have attempted to investigate the molecular mechanisms driving chemotherapeutic resistance in SCLC by whole‐exome sequencing (WES) and transcriptome sequencing. The recurrent samples showed repeated mutations and loss of heterozygote in WNT signaling regulators, including CHD8 and APC. Activation of WNT signaling through down‐regulation of APC could induce chemoresistance.^8^ Gardner et al. did not discover a mutation difference between newly treated and relapsed tumors in human SCLC patient‐derived xenografts (PDXs). EZH2‐mediated epigenetic reprogramming leading to SLFN11 silencing was identified as the mechanism of inducing chemotherapy resistance.^9^ While Drapkin et al. was unable to observe a correlation between SLFN11 expression and chemotherapy sensitivity in SCLC PDXs.^10^ The driving mechanism of chemoresistance in SCLC is still unclear and large genome‐wide studies are needed. All of the above studies were based on biopsy tissue or PDXs model and focused on post‐relapses SCLC which responded poorly to almost all rescue treatments. Further exploration of the genomic profiling of pre‐relapse residual tumors and eradication of the root of recurrence might help to ameliorate the current dilemma.^11^

In this study, we aimed to investigate the genomic profiling and molecular characteristics associated with chemoresistance by WES in surgically resected specimens of SCLC with limited disease (LD) after neoadjuvant chemotherapy. It provides reference for further elucidating the mechanism of drug resistance and relapse, as well as seeking for effective treatment.

## 2 METHODS

### 2.1 Patients' cohort

A retrospective analysis was performed on 416 patients with LD SCLC who received surgical treatment in Cancer Hospital of Chinese Academy of Medical Sciences from January 2006 to January 2016, including 40 patients who received neoadjuvant chemotherapy. A total of 19 patients with different efficacy of neo‐adjuvant therapy (NAC group, 12 cases of partial response [PR], 5 cases of stable disease [SD], and 2 of progressive disease [PD]) were screened for further genome studies following with the criterion (Figure S1): pathologically pure SCLC tissue, received two or more cycles of etoposide and cis‐platinum (EP)/carboplatin (EC) regimens (or combined with radiotherapy), sufficient tissue for DNA extraction and sequencing, complete follow‐up information. Other 10 SCLC patients with TNM pathological Stage I–II and received surgery combined with 4 cycles EP/EC adjuvant chemotherapy were enrolled as control group (chemotherapy naïve [CTN] group). Of which, five patients relapsed and died within 2 years and the other five patients survived for more than 5 years. Tumors of the above 29 patients and matched para‐tumor tissues were available. Pathological hematoxylin–eosin staining and immunohistochemistry sections were independently reviewed by two pathologists with more than 5 years of experience to confirm the diagnosis.

The detailed clinical data was obtained from medical records and follow‐up, including age at diagnosis, smoking history, Karnofsky Performance Status Scale score, clinical and pathological stage, clinical intervention, efficacy evaluation, and survival status. The clinical response to neo‐adjuvant treatment was evaluated based on computed tomography scans every 2 months during treatment and was classified as complete response (CR), PR, SD, or PD by using the standard RECIST 1.1 criteria.^12^ The objective response rate (ORR) = (CR + PR)/total cases × 100%. Pathological response evaluation for neo‐adjuvant therapy was based on the Miller & Payne grading system^13^ and was divided into three grades: Mild, Grade 1–2; moderate, Grade 3; severe, Grade 4–5. Recurrence‐free survival (RFS) was calculated from surgery to disease relapse, death, or the date of the last follow‐up. OS was defined as the start of neo‐adjuvant therapy in NAC group or surgery in CTN group until death from any cause or the date of the last follow‐up. And this study was approved by the medical ethics committee of the Cancer Hospital of the Chinese Academy of Medical Sciences (CAMS). All patients provided written informed consent.

### 2.2 Tissue preparation and DNA extraction

All specimens for WES were collected from surgically resected formalin‐fixed paraffin‐embedded (FFPE) tissues and 8 ums × 10 sections tumor and paired para‐tumor tissues were prepared for DNA extraction. Tumor cells were confirmed under microscope and microdissection was used to guarantee tumor content (proportion of tumor cell nuclei >50% and necrosis <30%). DNA was extracted using QIAamp DNA FFPE Tissue Kit based on manufacturer's instructions, and was quantified by Qubit (Life Technologies) and DNA integrity was examined by agarose gel electrophoresis.^14^ DNA extraction, library, sequencing, and information analysis were all performed by Beijing Novogene Biotechnology Co., Ltd.

### 2.3 Library preparation and WES

Agilent's liquid‐chip capture system was used to efficiently enrich human whole‐exon DNA, and the library preparation and capture experiments were conducted using the Agilent SureSelect Human All Exon kit according to manufacturer's instructions and optimized experiment operations. Genomic DNA were sheared randomly into 180–280 bp fragments by Covaris S220 sonicator and purified by AMPure SPRI beads (Agencourt). After the ends‐repair and A‐tailed, the DNA fragments were ligated indexing‐specific adapters. AMPure XP bead purification was used to remove unligated adaptors and PCR with SureSelect Primer and SureSelect Pre‐capture Reverse PCR primers were used to prepare DNA libraries. The library with specific indexes was pooled and hybridized with a biotin‐labeled probe. Then, a total of 334,378 exons from 20,965 genes were captured using a magnetic bead with streptomycin and were linearly amplified by PCR for quality inspection. The library was diluted to 1 ng/μl and the insert size was detected using Agilent 2100. Q‐PCR was used to accurately quantified the effective concentration of the library (>2 nM) to ensure library quality. The qualified specimens were sequenced on Illumina HiSeq4000 sequencing platform (Illumina) according to manufacturer's instructions for paired‐end 150 bp (PE150) reads (Novogene). The target sequencing depth of tumor samples and normal tissues were 200× and 100×, respectively.

### 2.4 Data quality control and bio‐information analysis

The Sequenced Reads that converted from raw image files and obtained from high‐throughput sequencing by Base Calling analysis are called Raw Data and stored in FASTQ (fa for short) format. Raw data was filtered as following: Reads with adapter deletion; Delete reads containing more than 10% of unrecognized base information; and removal of paired reads when low‐quality (less than 5) bases of single‐ended reads exceeded 50% in length. The clean data was aligned to the reference genome (B37) by Burrows‐Wheeler Aligner and Samblaster to obtain the initial BAM file.^15^ The BAM file was then filtered by the SAMtools, Picard (http://broadinstitute.github.io/picard/) and GATK, local realignment, and recalibrated of base quality to generate the final BAM file for computation of sequence coverage and depth.^16^, ^17^

Somatic single nucleotide variants (SNV) and InDel were detected by MuTect and Strelka,^18^, ^19^ somatic copy number variation (CNV) was detected by Control‐FREEC or VarScan.^20^ ANNOVAR software was applied to annotate Variant Call Formats as following^21^: Refseq and Gencode were used to annotate gene structure, gene type, and genomic features of variant locus; dbSNP, 1000 human genome SNP database, Hapmap database, Catalog of Somatic Mutations in Cancer (COSMIC), and esp6500 variation database were adopted to screen for non‐synonymous somatic mutations; SIFT, PolyPhen, MutationAssessor, LRT, and other methods were performed to predict the impact of nonsynonymous mutations on diseases/tumors^22^, ^23^; GO Database, KEGG, Reactome, Biocarta, PID, etc. were performed to annotate the functions of signal transduction and metabolic pathway. MuSic and PathScan software were selected to screen significantly mutant genes and analyze pathway enrichment of significantly mutant genes, respectively. Control‐FreeC was applied for detection of somatic CNV and GISTIC software was performed to assess the reproducibility of CNV.^20^ Mutation Relation Rest (MRT) was analyzed by MuSiC software.^24^ The significantly mutant genes were compared with NovoDrug and NovoDR database to predict the targetable genes and resistance‐related mutations.

### 2.5 Mutational spectrum and signature analysis

Somatic SNV mutational spectrum and signature analysis are helpful to understand the characteristics of tumor at the level of point mutation. Point mutations were divided into six types of variation. Mutational spectrum analysis was performed to analyze the preference of point mutations in tumors and the similarity between different samples by clustering the tumor samples and point mutation types which were calculated by the number of point mutations. The mutational signature analysis was based on the number of 96‐point mutations in each tumor sample, and the non‐negative matrix factorization (NMF) method was performed to extract the mutational features of somatic point mutations. Comparing the mutational signature of tumors with 30 known signatures in COSMIC database could reflect the physical, chemical, or biological processes of somatic mutations in cancer.

### 2.6 Data filter for FFPE samples

Based on the previous description, we strengthened the filtering conditions to exclude artificial mutations in FFPE samples, as shown below: Quality Control was same as above; Milup software was applied to calculate the preference; The mutect parameters were adjusted as following: (1) Determine the random error rate using control samples; (2) reduce the preference of mutect software; (3) expand Indel's distance; (4) filter similar reads (read distance was determined based on variance test of all reads); (5) mutation load LOD of mutect was adjusted from 6.3 to 1.8 to improve accuracy and reduce sensitivity; (6) remove the cases when chain‐preferred C>T mutation ratio >5:1 and the sites not counted in the Milup files; (7) DP ≥10; DV >3 (reads with different start and end sites); (8) retain the site of vaf ≥0.1 when variable reads ≥6; otherwise retain site of vaf ≥0.15 when variable reads = 4 or 5; (9) filter other mutation types besides C>T and site of variable reads ≤2 in same direction.

### 2.7 Statistical analysis

In this study, statistical significance of differences observed between the count data groups were determined by *t* test or ANOVA analysis when comparing countious variables, and the *χ* ^2^ test when comparing frequencies. The Wilcoxon rank‐sum test was used to compare the difference of clinical and pathologic parameters between NAC and CTN groups. Survival curves were estimated by using the Kaplan–Meier method, and the log‐rank test. The multiple Cox's proportional hazard model was used for multivariate analyses with the variables from the univariate analysis to predict the hazard rates for RFS and OS. The statistical significance level was defined as two‐sided *p* < 0.05. All statistical analyses were performed with SPSS19.0 software (IBM Corp.).

## 3 RESULTS

### 3.1 Whole genomic profiling analysis

**FIGURE 1:**
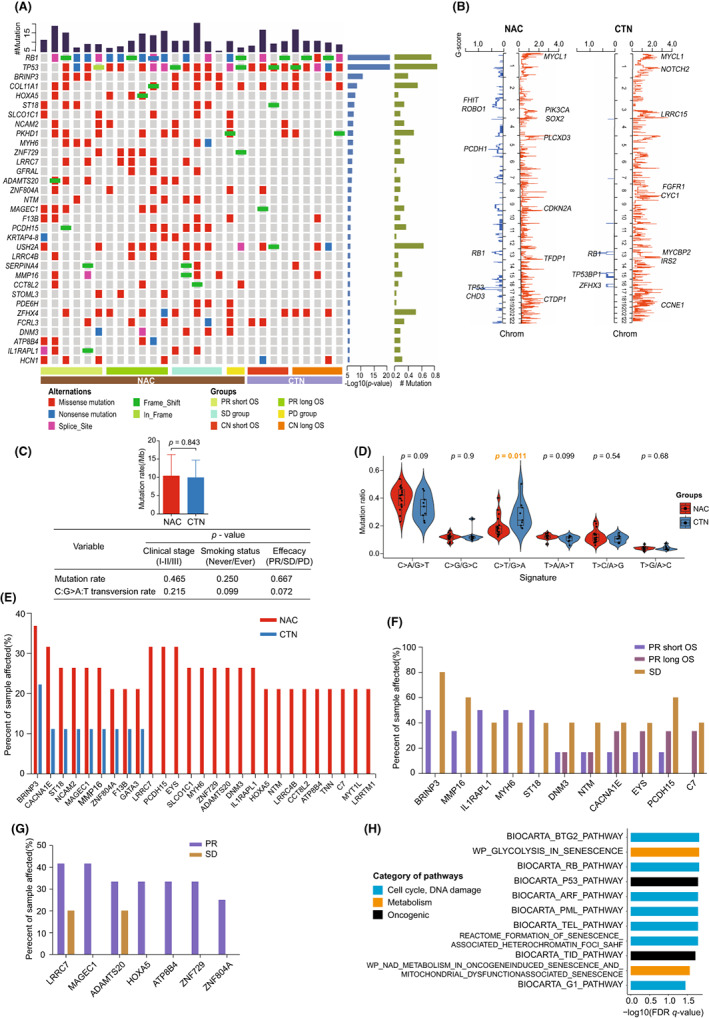
Genomic alterations in small cell lung cancer with neo‐adjuvant chemotherapy. (A) Comparison of significantly mutant genes between neo‐adjuvant chemotherapy (NAC) and chemotherapy naïve (CTN) group. Samples are arranged based on groups listed at bottom. Mutation rates are listed on the top. Mutation types of significantly mutant genes are annotated for each sample according to the color panel below the image. The differential significance (*p* value) and patient‐affected frequency for each gene are plotted on the right panel in turn. (B) Comparison of copy number variant (CNV) alterations between NAC and CTN groups. Red and blue are plotted as amplifications and deletions of chromosomal regions, respectively. (C) Upper, comparison of mutation rate between two groups, using independent *t* test. Lower, analysis of the effects of clinical stage, smoking status and different efficacy on mutation rate and signature in NAC group. (D) Comparison of mutation signature between two groups, using nonparametric tests. (E) Ratio of sample affected of 27 frequently mutant genes in NAC group. (F) Eleven genes are recurrent in both PR and SD group. (G) Seven genes are more frequently mutant in PR group than in SD group. (H) The frequently mutant genes in NAC group are enriched in 11 pathways. PR, partial response; SD, stable disease.

The efficacy of neo‐adjuvant therapy combined with surgery was evaluated in all 40 patients (Tables S1 and S2), and the prognosis was similar among patients with residual tumors regardless of initial response (PR or SD). We hypothesized that genetic alterations in residual tumors were important factors affecting survival. And WES was used to explore the genomic profiling associated with chemoresistance from mutational signature, CNV, significantly mutant genes and differential genes (Figure S2). The basic clinical characteristics were consistent between NAC and CTN group (Table 1). Excluding one hypermutated sample, 28 paired samples with tumor and para‐tumor tissue were enrolled into the following analysis (Table S3). The average sequencing depth of tumor and normal samples were 203.04× (ranged from 150 to 310×) and 117.86 × (ranged from 69 to 247×). Average Q30 were 91.19% (ranged from 87.40 to 95.66%) and 90.34% (ranged from 85.98 to 95.90%). Fraction of target‐covered with at least 10x were 99.54% (ranged from 98.81 to 99.82%) and 98.94% (ranged from 97.63 to 99.74%). Mutation analysis within 28 paired samples identified 31,104 somatic mutations in 7045 genes, of which 36.3% were protein‐altering SNV (Table S4). The ratio of non‐synonymous and synonymous SNV was 3.20:1, which was higher than the previous study.^25^ The mean protein‐altering non‐synonymous SNV of whole cohort was 307.46 (ranged from 58 to 689) / per sample and mean non‐synonymous mutation rate was 10.25 per mega base (/Mb). There was no significant difference in mutation rate between NAC and CTN group (10.39/Mb vs. 9.94/Mb, *p* = 0.843). The smoking history or clinical stage of the tumors were not correlated with protein‐altering non‐synonymous mutation rate (*p* = 0.250, 0.450) in NAC group. No significant difference of non‐synonymous mutation rate was observed between different efficacy groups (*p* = 0.667, Figure 1C).

**TABLE 1:** Basic clinical characteristics of 29 patients enrolled in this study

Variables	Neo‐adjuvant chemotherpy (*n* = 19)	Chemotherapy naïve (*n* = 10)	*p*‐Value^a^
Age			0.449
≤60	9	3	
>60	10	7	
Gender			0.675
Male	14	6	
Female	5	4	
Smoking			0.694
Yes	11	7	
No	8	3	
KPS			0.268
≥90	15	10	
<90	4	0	
Weight lost			0.408
Yes	3	1	
No	16	9	
Tumor location			0.114
Left	5	6	
Right	14	4	
Surgical procedures			0.134
Pulmonary lobectomy	14	10	
Unpulmonary lobectomy	5	0	
Adjuvant chemotherapy			0.633
≥4 cycles	14	9	
<4 cycles	5	1	
Adjuvant radiotherapy			0.633
Yes	5	1	
No	14	9	
PCI			0.414
Yes	8	2	
No	11	8	
Clinical stage			0.290
I	11	7	
II	4	3	
III	4	0	
Pathological stage			0.166
I	7	7	
II	7	3	
III	5	0	
Brain metastasis			0.431
Yes	7	2	
No	12	8	

Abbreviations: KPS, Karnofsky performance score; PCI, Prophylactic cranial irradiation.

^a^ Fisher exact test.

### 3.2 Recurrent somatic mutations in NAC group

To further explore the recurrent somatic mutations, we identified 33 and 3 significantly mutant genes (*q* < 0.05) with MutSigCV method in NAC and CTN groups, respectively (Figure 1A; Table S5). *TP53* and *RB1* were the most significant mutant genes with similar frequency (78.95% vs. 100%, 78.95% vs. 66.67%) between two groups. In addition to *TP53* and *RB1*, the frequent mutation genes in the NAC group were involved in proliferation and apoptosis (*PKHD1*, *GFRAL*, *ADAMTS20*, *BRINP3*), pathway regulation and signaling conduction (*COL11A1*, *MYH6*, *PCDH15*, *IL1RAPL1*, et.al), epithelial mesenchymal transition (*HOXA5*, *NTM*), DNA transcriptional regulation (*ST18*, *ZNF729*, *ZFHX4*), CAMs signaling pathway (*NCAM2*, *LRRC4B*), metalloproteinase activity (*MMP16*) and immune cell activation (*FCRL3*, *ATP8B4*, *LRRC7*, *SERPINA4*).

**FIGURE 2:**
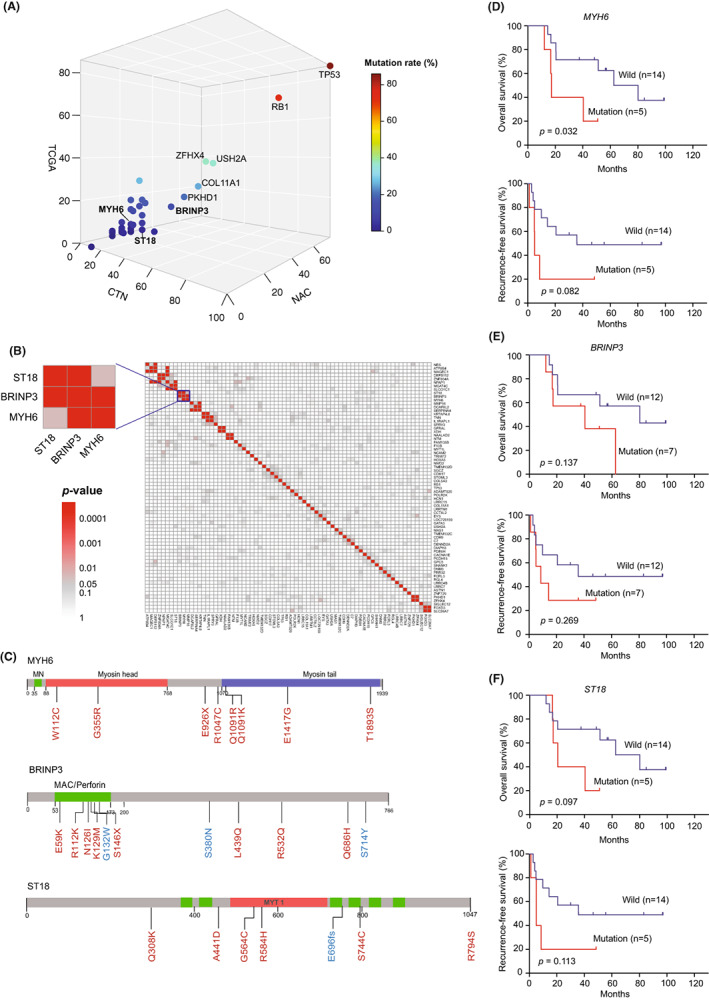
The frequently mutant genes in neo‐adjuvant chemotherapy (NAC) group are interactive and might be associated with patients' survival. (A) 3D diagram denoted the mutation rate of frequently mutant genes in NAC group. (B) Mutation Relation Rest (MRT) demonstrate mutually synergetic relationship between genes frequently mutated in NAC group. (C) Schematics exhibit the distribution of *MYH6*, *BRINP3* and *ST18* somatic mutations on protein domains. Red font existed in NAC, and blue font existed in chemotherapy naïve (CTN). (D–F) Survival analysis of patients with limited diseases small cell lung cancer in NAC group based on mutant status of *MYH6*, *BRINP3* and *ST18*. Using log‐rank test.

Function‐acquired mutations often cluster in specific protein regions which endow cancer cells with selective growth advantage and undergo positively selective pressure during tumor development. We performed the OncodriveCLUST analysis^26^ to screen the clustered mutations. The previously reported clustered mutations in *EP300* and *CREBBP* ^27^ were not found in this study. But we discovered that the locally clustered mutations occurred in *BRINP3*, *MYH6*, *COL11A1*, *PKHD1*, and *SYNE1* (Figure 2C and Figure S3a). Of which, mutation clustering of *MYH6*, *ZNF729*, *LRRC7*, and *PCDH15* only occurred in NAC group, which were involved in WNT signaling pathway, DNA transcription, protein binding, and calcium transport, respectively. However, the function of these genes in tumor remains unclear.

**FIGURE 3:**
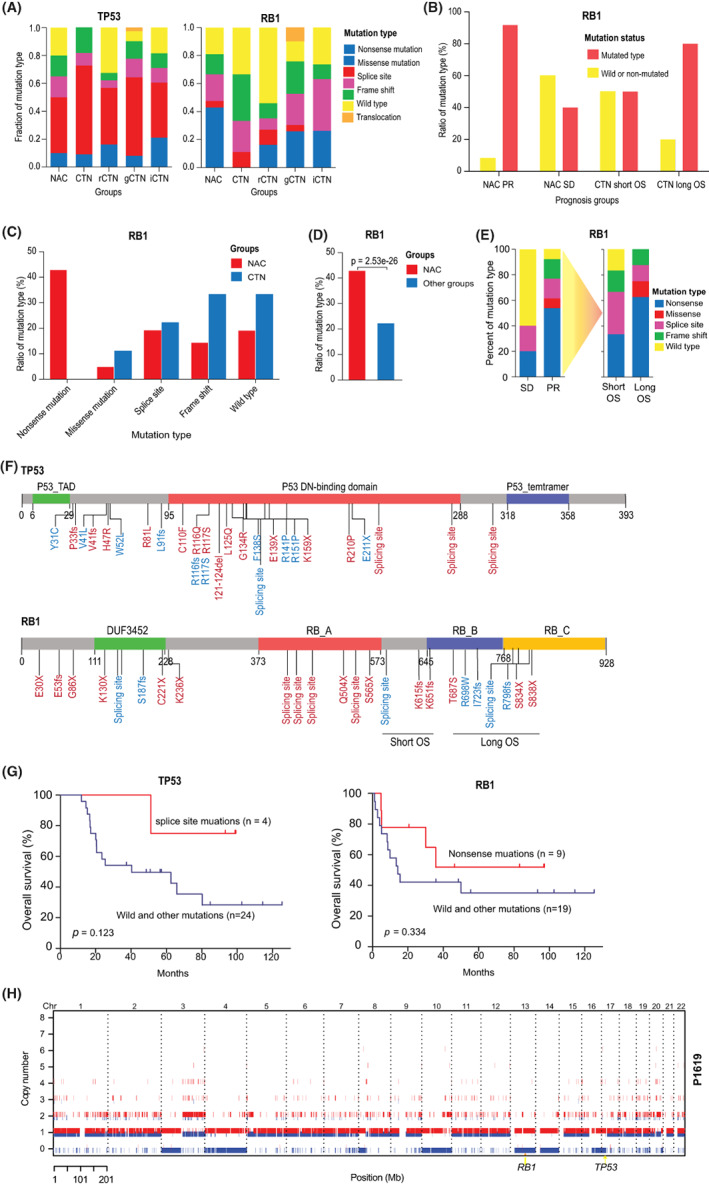
*RB1* mutation types are different in patients with neo‐adjuvant chemotherapy. (A) Comparison of *TP53* and *RB1* mutation types fraction in neo‐adjuvant chemotherapy (NAC), chemotherapy naïve (CTN), and other three studies. rCTN, gCTN, and iCTN studied by Rubin. George and Iwakawa et al., respectively. (B) Comparison of the relative ratio of mutant *RB1* and non‐mutant types in NAC and CTN groups with different efficacy and survival. (C) Comparison of *RB1* mutation types between NAC and CTN groups. (D) The ratio of *RB1* nonsense mutations in NAC group is significantly higher than other studies. Using Fisher exact test. (E) Comparison of *RB1* mutation types between stable disease (SD) and partial response (PR) groups with different survival in NAC group. (F) Schematics exhibit the distribution of *RB1* and *TP53* somatic mutations on protein domains. (G) Survival analysis of *TP53* and *RB1* with different mutation type. Using log‐rank test. (H) Representative schematics exhibit the copy‐neutral LOH occurring at the region of chromosome 13 (harboring *RB1*) and chromosome 17 (harboring *TP53*).

We integrated treatment information from PG_FDA, PharmGKB, and MyCancerGenome databases to look for  targetable mutations (Table 2). And we found that *KIT*, *ROS1*, *ALK*, *RET*, and *KRAS* mutations presented in eight cases, but no *EGFR* mutation was detected in NAC group. Of which, one patient (patient P1615) harbored both *KRAS* and *ROS1* mutations. Inversely, the *EGFR*, *ROS1*, *RET*, and *KIT* mutations were detected in five cases of CTN group. All the results suggested that some genetic subtypes might benefit from the targeted therapy.

**TABLE 2:** List of targetable genes in neo‐adjuvant chemotherapy and chemotherapy naïve group

Group	Genes	Samples	Variant	cDNA changes	Protein changes
Neo‐adjuvant chemo	*KIT*	P1610	Missense_Mut	c.C2900A	p.S967Y
		P1618	In_Frame_Del	c.1253_1255del	p.418_419del
	*ROS1*	P1616	Missense_Mut	c.A4135G	p.M1379V
		P1615	Missense_Mut	c.C6548A	p.P2183Q
		P1613	Missense_Mut	c.G4354A	p.V1452M
		P1602	Missense_Mut	c.G295T	p.A99S
	*RET*	P1609	Nonsense_Mut	c.A3178T	p.K1060X
	*ALK*	P1617	Missense_Mut	c.C737A	p.T246N
	*KRAS*	P1615	Missense_Mut	c.G35A	p.G12D
Chemotherapy naïve	*EGFR*	P1627	Missense_Mut	c.G1562A	p.R521K
	*ROS1*	P1625	Missense_Mut	c.G5525C	p.S1842T
	*RET*	P1627	Missense_Mut	c.G319A	p.E107K
		P1624	Frame_Shift_Del	c.2534delC	p.A845fs
	*KIT*	P1620	In_Frame_Del	c.1253_1255del	p.418_419del

### 3.3 Recurrent CNVs of SCLC patients

To investigate the recurrent CNVs in SCLC patients, the frequency of minor alleles (BAF) at CNV and SNV loci in somatic cells was analyzed by Control‐FREEC software, and the reproducibility was evaluated by GISTIC software (Figure 1B). In line with previous studies, we observed local amplification concentrations in CTN group were 1p32.3–1p34.3 (including *MYCL1*), 8p21.1 (including *FGFR1*), 13q14.3–13q34 (including *MYCBP2*, *IRS2*), and 19q12 (including *CCNE1*), accompanied by focal deletions on 13q14.2 (including *RB1*) and 15q14 (including *TP53BP1*). In NAC group, we confirmed local amplifications on 3q13.32 (including *SOX2*, *PIK3CA*), 1p34.2 (including *MYCL1)* and recurrent deletions on 3p14.2 (including *FHIT* and *ROBO1*), 13q13.3 (including *RB1*), and 17p13.1 (including *TP53*) regions. We also discovered the copy‐number gain within 9p21.3 containing *CDKN2A* locus, a periodic homozygous deletion by George et al.^27^

There are overall more CNV deletions in NAC group but amplifications in CTN group. Compared with NAC group, local amplifications recurred more frequently on 1q21.2, 6q27, 8q24.23, 8q24.3, 9p13.2, 17q21.2, 17q22, 17q25.1, and 19q13.11 in CTN group. Multiple genes were located on 1q21.2, such as *NOTCH2*, *NTRK1*, *DDR2*, and *DNM3*. Of which, *PAX5* was located on 9p13.2, and *SOX9* and *MYCBPAP* were located on 17q22. However, NAC group demonstrated more deletions than CTN group, especially on 5q31.3 and 17p13.1, and the latter was the loci of *TP53* gene. These results indicated that the concurrent mutations and deletions of *TP53* gene happened more frequently in NAC group. Patients with *TP53* deletion demonstrated improved RFS and OS, even though it did not reach the statistical significance (*p* > 0.05).

### 3.4 Different mutation signatures in SCLC

To further explore the difference of mutation signatures between NAC and CTN group, we detected the mutation signatures in all SCLC patients. As a result, we discovered that C:G>A:T transversions were the predominant point mutation type in whole cohort, accounting for 38.2% and followed by C:G>T:A for 22.2%. And the somatic mutation signatures were displayed by NMF method, and clustered with 30 known signatures registered in COSMIC database. The overall mutation signatures were highly consistent with Signature 4, which is a pattern of heavy smoking. The smoking history or clinical stage were not associated with mutation signature in NAC group (*p* = 0.099, *p* = 0.215, Figure 1C). The signatures between NAC and CTN groups were inconsistent. The mutation signatures of NAC group presented more similar across samples and obviously high fraction of C:G>A:T transversions (*p* = 0.090), but more prevalent C:G>T:A transitions (*p* = 0.011) in CTN group (Figure 1D). Furtherly, these mutation signatures displayed a relatively high fraction of T:A>A:T transversions (*p* = 0.045) in patients with SD than PR in NAC group. And then hierarchical clustering of six mutation signatures divided our cohort into two groups. The result showed that most patients with PR and SD in NAC group presented different mutation signatures from CTN group, but those with PD status in NAC were consistent with CTN group (Figure S3b).

### 3.5 Differential genes analysis identified the candidate genes associated with resistance and survival

To explore the differential genes associated with drug resistance and survival, we determined the significance *q*‐value <0.1 between NAC and CTN group (Figure S4a). In addition to *TP53* and *RB1*, the significantly mutated genes *USH2A*, *ZFHX4*, *COL11A1*, *PKHD1*, *SHANK1*, *FCRL3*, and *HCN1* in NAC group also recurred in CTN group with similar mutation rates. These genes were also frequently mutated in CTN patients of three comprehensive genomic studies^27^, ^28^, ^29^ (Figure S4b,c; Table S6). However, another six genes (*TBX10*, *CYBB*, *SV2C*, *KCNJ14*, *ZSCAN4*, and *UGT2B11*) were frequently mutant in CTN group, but not in NAC group. This suggested that they might be involved in the initiate and maintenance of SCLC.

Next, a total of 27 frequently mutated genes in NAC group affected more than 20% of these samples (Figure 1E), which might be related to chemotherapy resistance. Eleven genes including *BRINP3*, *MYH6*, *ST18*, and *PCDH15* recurred in both PR and SD groups, which might be involved in the primary resistance (Figure 1F). And the other seven mutant genes (e.g., *MAGEC1*, *HOXA5*, and *ZNF729*) frequently occurred in the PR group, which might be correlated with developed resistance (Figure 1G). Integrated pathway analysis revealed that the frequently mutated genes in NAC group were involved in cell cycle, metabolic reprogramming, and oncogenic signaling pathways such as BTG2 pathway, glycolysis in senescence and P53 pathway (Figure 1H). Furtherly, we discovered *BRINP3*, *MYH6*, and *ST18* mutations were high‐mutated in NAC group compared with CTN group and TCGA database (Figure 2A). And then, to investigate the relationship among these significantly mutated genes, we performed MRT analysis (Figure 2B). It demonstrated that *BRINP3* mutation co‐occurred with *MYH6* and *ST18*, while was mutually exclusive with *ZNF729*.

Furthermore, we found that multiple genes co‐occurred in PR and SD groups were associated with patients' survival (Figure 2D–F). The OS rate of patients with *MYH6* mutation was significantly lower than that of wild type patients (*p* = 0.032). And *BRINP3*, *ST18*, and *IL1IAPL1* were associated with poor OS, while *PCDH15* might improve survival (Figure S4d). Patients with *MYH6* and *ST18* mutations also had shorter RFS, while patients with *C7* mutation had relatively longer RFS. The recurrent mutations occurred only in PR group were not associated with survival. And multiple Cox regression including clinical parameters and mutant genes demonstrated that the above mutated genes were not dependent factors in predicting the prognosis of SCLC patients.

Then, we evaluated the relationship among mutated genes, clinical parameters, mutation rate, and mutation signature (Table 3). The results showed that mutations of *BRINP3*, *MYH6*, *PCDH15*, *LRRC7*, and *FCRL3* occurred only in patients with mild pathological response, while mutations of *CCT8L2* and *ATP8B4* were mostly present in patients with moderate and severe response. And *LRRC7* and *MMP16* mutations were more frequent in late stage, but *EYS*, *C7*, and *SLCO1C1* mutations in early stage. *EYS*, *ZNF729*, and *C7* mutations reoccurred in smokers but *ST18* in non‐smokers. Also, the non‐synonymous mutation rates of *PCDH15*, *F13B*, *CCT8L2*, *LRRTM1*, and *MAGEC1* genes were significantly higher than those with wild‐type patients. And patients harboring *GATA3* and *MAGEC1* mutations had significantly higher C:G>A:T transversion rate than those with wild type.

**TABLE 3:** List of significantly correlated genes with clinical parameters in patients treated with neo‐adjuvant treatment and surgery

Variable	*BRINP3*	*PCDH15*	*CCT8L2*	*LRRC7*	*SLCO1C1*	*ZNF729*	*MAGEC1*	*F13B*	*LRRTM1*	*GATA3*	*ST18*	*MYH6*	*FCRL3*	*MMP16*	*EYS*	*C7*
	*p* Value	*p* Value	*p* Value	*p* Value	*p* Value	*p* Value	*p* Value	*p* Value	*p* Value	*p* Value	*p* Value	*p* Value	*p* Value	*p* Value	*p* Value	*p* Value
Gender (male vs. female)	1	1	1	1	0.57	0.257	0.084	0.037	0.272	1	0.084	0.397	0.603	0.603	0.111	0.258
Age (≤60 year vs. >60 year)	0.057	1	0.303	1	1	0.141	0.628	0.582	1	0.582	0.184	0.184	0.184	0.556	0.259	0.333
Smoking status (ever vs. never)	1	0.319	0.603	1	1	0.045	0.603	0.262	1	0.603	0.071	0.336	0.336	0.664	0.153	0.085
Neo‐ajuvant effecacy (PR/SD/PD)	0.053	0.22	0.563	0.407	0.707	0.263	0.138	0.563	0.723	0.723	0.547	0.547	0.707	0.121	0.698	0.416
Pathological evaluation (mild vs. moderate/severe)	0.044	0.109	0.071	0.109	1	0.262	0.262	0.557	0.557	0.557	0.48	0.111	0.111	0.48	0.257	0.627
Tumor location (left vs. right)	0.305	1	0.272	1	1	0.57	0.57	1	0.272	0.272	0.397	0.397	0.172	0.397	0.52	0.272
Clinical stage (I–II vs. III)	0.377	1	1	1	0.045	0.338	1	0.603	1	0.603	0.664	0.336	0.664	0.071	0.153	0.085
Pathological stage (I–II vs. III)	1	0.262	0.53	0.017	1	0.172	0.57	0.53	1	1	0.603	0.397	0.603	0.397	0.111	0.258
Mutation rate	0.893	0.025	0.026	0.799	0.315	0.304	0.024	0.024	0.009	0.177	0.333	0.522	0.858	0.328	0.245	0.186
C:G>A:T transversion rate	0.838	0.078	0.438	0.894	0.302	0.399	0.015	0.22	0.187	0.038	0.174	0.521	0.721	0.76	0.446	0.316

Abbreviations: PD, progressive disease; PR, partial response; SD, stable disease.

### 3.6 *TP53* and *RB1* loss types might be correlated with patients' efficacy and survival

In our cohort and another three previous studies,^27^, ^28^, ^29^ *TP53* mutation types occurred with similar frequency (Figure 3A). There was no preference for the distribution of different mutation types in the amino acid sequence (Figure 3F). Compared with other mutations, the survival rate of patients with *TP53* splicing site mutation was improved, but the difference was not significant (*p* = 0.123, Figure 3G).

Previous study has shown that *RB1* mutation status is a good predictor of efficacy and prognosis in SCLC.^30^ In our study, mutant *RB1* was more frequent in CTN long OS group (4/5) and NAC PR group (Figure 3B). It suggested that mutant *RB1* might be correlated with good efficacy and survival. However, Kaplan–Meier analysis showed that *RB1* mutation was not associated with patient survival. The mutation types of *RB1* were significantly different in two groups. Nonsense mutation occurred frequently in NAC group (9/19, 47.4%), but none was observed in CTN group (Figure 3C). We also explored *RB1* mutation types of SCLC patients with CTN in previous three studies.^27^, ^28^, ^29^ And we found that nonsense mutation accounted for 12.6%–26.3%, which was significantly lower than that of NAC group (*p* < 0.001, Figure 3D), which was mainly distributed in the PR long OS group (5/6, Figure 3E). Notably, one patient (patient P1605) in PR group with three types of *RB1* mutations (nonsense mutation, missense mutation, and splicing site) obtained PR after one cycle of EP regimen and survived for more than 8 years. Compared with other types of patients, patients with nonsense mutations had improved survival, although the differences were not significant (Figure 3G). These results suggested that *RB1* nonsense mutation might be associated with better efficacy and survival. Furthermore, mutations occurring in a domain following the amino acid sequence might be correlated with good survival (Figure 3F).

Interestingly, in one patient (patient P1619) lack of *TP53* and *RB1* mutations, we observed large scale copy‐neutral losses of heterozygosity (LOH) in regions of 13 and 17 chromosomes harboring *RB1* and *TP53*, respectively (Figure 3H). In addition, copy‐neutral LOH and LOH were observed in the high ploidy of the chromosome region containing *RB1* in the other four samples without *RB1* mutation, except for two samples which were difficult to determine due to poor quality.

## 4 DISCUSSION

In SCLC patients, residual tumor after the initial response leads to eventual drug resistance and relapse. It is of great significance to explore the genetic background and available measures of eradicating them. However, the lack of tissue samples severely restricts the development of research. In the current study, we first investigated the genomic profiling of residual tumors in SCLC.

Tumor mutation burden was a prognosis and ICIs efficacy indicator in SCLC.^31^, ^32^ There was no difference in protein‐altering SNV and mutation rate between NAC and CTN group. In the present study, in addition to the recurrent deletion of regions harboring *FHIT* and *RB1*, amplifications of *MYCL1* and *SOX2* were confirmed.^33^, ^34^ Xiaohui Ni et al. confirmed that circulating tumor cells (CTCs) from an individual patient exhibited reproducible CNV patterns,^35^ and CNV patterns remained constant during treatment. Different from the study by Xiaohui Ni, NAC group demonstrated more deletions than CTN group, especially on 5q31.3 and 17p13.1. Concurrent mutations and deletions of *TP53* gene happened more frequently in NAC group, and correlated to improved survival. And the relationship between CNV pattern and treatment needed to be further validated in more SCLC samples.

Mutation signature analysis divided patients into two groups. Patients in PR and SD groups had a different mutation signatures from those CTN patients, whose signatures were more similar to patients with disease progression. And the mutant signatures were more different between patients in CTN group than NAC groups. It suggested that CTN patients had more obvious heterogeneity compared with the residual tumors. We speculated that the mutation signatures might be correlated with disease progression and could be used as a marker to help monitor disease evolution and distinguish different subtypes. However, large samples were still needed for further confirmation.

*TP53* and *RB1* were the most significant mutation genes in SCLC. Missense mutation was frequent in *TP53* and affected DNA binding domain and region between transactivation motif. Mutation occurred in splice sites could cause tremendous protein‐damaging events and might correlate with improved survival.^36^ We also observed that patients with *TP53* splice site mutation improved survival compared with other mutation types. And mutant *RB1* was recurrent in NAC PR group and CTN group with long OS. It is consistent with the study by Dowlati et al. confirming that mutant *RB1* predicts good efficacy and prognosis.^30^ With the exception of two samples that were difficult to be diagnosed due to poor quality, the other samples lacking *RB1* mutants were observed with the copy‐neutral LOH or LOH at higher ploidy on 13 chromosome harboring *RB1* gene. In one patient (patient P1619) simultaneously lack of *TP53* and *RB1* mutation, copy‐neutral LOH were observed on 13 and 17 chromosomes harboring two genes, respectively. That means that the inactivation of *TP53* and *RB1* were obligatory in SCLC.^27^ The “wild type” *RB1* was associated with poor efficacy and prognosis in the previous study by Dowlati et al.^30^ Because of limitation of targeted sequencing, CNVs could not be observed. In fact, patients with “wild type” *RB1* might frequently occur LOH of CNV, which brought about a tremendous devastation associated with worse response and survival compared with inactive mutations.

Different from *TP53* missense mutation, *RB1* was frequently altered due to frame‐shift deletion and splicing site mutation in CTN group. However, the inactive mutations were significantly different in NAC group, in which nonsense mutations were the most frequent types. Nonsense mutations in *RB1* were also recurrent in PR group and associated with favorable survival. The change of *RB1* mutant status might be correlated with the CR induced by NAC.^37^ It suggested that *RB1* nonsense mutation and change of mutant status might be good predictors of efficacy and prognosis.

Of 27 frequently mutated genes in NAC group, 11 genes were recurrent in PR and SD groups, and multiple genes were associated with survival. Seven mutant genes only occurred in PR group. We speculated that tumor cells harboring 11 genes were the mainly resistant clone associated with the chemo‐resistance and survival while not those in PR group only. *BRINP3*, *MYH6*, and *ST18* mutations were locally clustered and mutually synergetic in many patients with mild response to induction chemotherapy, and were recurrent in PR and SD groups with short RFS and OS. They might cooperate in drug resistance and rapid progression of SCLC. *BRINP3* participates in the differentiation of neuronal stem cells and overexpression was involved in the tumorigenesis of pituitary gonadotropin by increasing tumor cell proliferation, invasion, and metastasis.^38^ And *MYH6* was involved in the differentiation of cardiac stem cells by activating the WNT and BMP signaling pathway. Activation of WNT signaling through APC knockdown induces chemoresistance.^8^Recently, it was discovered as a carcinogenesis gene and closely related to survival.^39^, ^40^ Xiaohui Ni et al. analyzed the differences in CTCs pre‐ and post‐ chemotherapy by single cell technology, and disvovered an increased mutant frequency of *MYH7*, a same series of gene with *MYH6*, might participate in disease progression by “ATP binding” pathway.^35^ The *ST18* gene has been proposed to act either as a tumor suppressor or as an oncogene in different human cancers and correlated with poor outcomes.^41^ It was down‐regulated in breast cancer, and the over‐expression suppressed the colony formation.^42^ *ST18* was also considered as an oncogene. *ST18* overexpression was associated with tumor progression and metastasis and contributed to the determination of small residual diseases (MRD). *ST18* knockdown resulted in an impaired cell proliferation and induction of apoptosis.^43^, ^44^, ^45^

*ADAMTS20* and *IL1RAPL1* mutations were mutually synergetic and associated with poor survival. They took part in proteinaceous extracellular matrix and voltage‐gated calcium channel signaling pathway, respectively.^46^, ^47^ *CACNA1E* was mutually synergetic with *PCDH15* and was also involved in calcium channel activity.^48^ Calcium channels were closely related to tumor cell proliferation, differentiation, metastasis, and resistance. Related genes were lower expressed in multiple tumors like lung cancer and breast cancer, and they were associated with chemotherapy resistance.^11^, ^49^ *LRRTM1* and *LRRC4B* were involved in the negative regulation of JAK–STAT cascade, negative regulation of protein kinase activity, and cytokine‐mediated signaling pathway. The inhibitors of cytokine signaling pathway were related to the malignancy and resistance although the specific mechanism was still elusive.^50^

In our study, we explored the genomic profiling of residual tumors after induction treatment. There was no difference in protein‐altering non‐synonymous SNV and mutation rate between NAC and CTN groups. NAC group demonstrated more CNV deletions than CTN group. The mutation signatures were obviously different. *TP53* and *RB1* mutations were the most significantly genetic alterations, and *RB1* nonsense mutations was dominated in NAC group associated with relatively good survival. Common mutated genes such as *BRINP3*, *MYH6*, *ST18*, and other frequently mutant genes in PR and SD groups might be involved in resistance and related to survival.

In conclusion, our study was the first investigation of genomic profiling of residual tumors after neo‐adjuvant treatment by WES in patients with LD SCLC. We systematically revealed the possible genomic background associated with resistance and survival. It provided important reference for clarifying the mechanism of resistance and relapse and seeking for treatments in SCLC. It is a pity that there are certain limitations in our study. First, this is a retrospective study with small samples, some results are still needed to be validated in larger samples; Second, FFPE samples brought about more artificial mutations, higher false positive rate, and inaccurate CNV results compared with fresh samples; Third, because of high heterogeneity in SCLC, the comparison between pre‐ and post‐ treatment from a same patient will provide more reliable results. Generally, our genomic sequencing analysis could obtain the common driver mutations that playing more important role in carcinogenesis, thus make target drugs selection more precise and effective for SCLC patients.

## AUTHOR CONTRIBUTIONS

Conceived and designed the experiments: Lvhua Wang, Jiangyong Yu. Performed the experiments: Jiangyong Yu, Shuangtao Zhao, Lihong Wu. Analyzed the data: Jiangyong Yu, Shuangtao Zhao, Zhe Su, Chengli Song, and Nan Bi. Contributed sample collection and processing, clinical data collection: Jingbo Wang, Nan Bi. Contributed to the writing of the manuscript: Jiangyong Yu, Shuangtao Zhao, Lvhua Wang.

## CONFLICT OF INTEREST

The author(s) declare that they have no competing interests.

## ETHICS STATEMENT

The study was approved by the medical ethics committee of the Cancer Hospital of the Chinese Academy of Medical Sciences (CAMS). All patients provided written informed consent.

## Supporting information


Figure S1.

Figure S2.

Figure S3.

Figure S4.

Table S1.

Table S2.

Table S3.

Table S4.

Table S5.

Table S6.

Figure‐captions.

## Data Availability

Some or all data, models, or code generated or used during the study are available from the corresponding author by request.
