# Supplementary figures and images for: Whole exome analysis reveals the genomic profiling related to chemo‐resistance in Chinese population with limited‐disease small cell lung cancer
Source: Cancer Med. 2022 Jun 23;12(2):1035–50. doi: 10.1002/cam4.4950 (PMC9883427; doi:10.1002/cam4.4950)

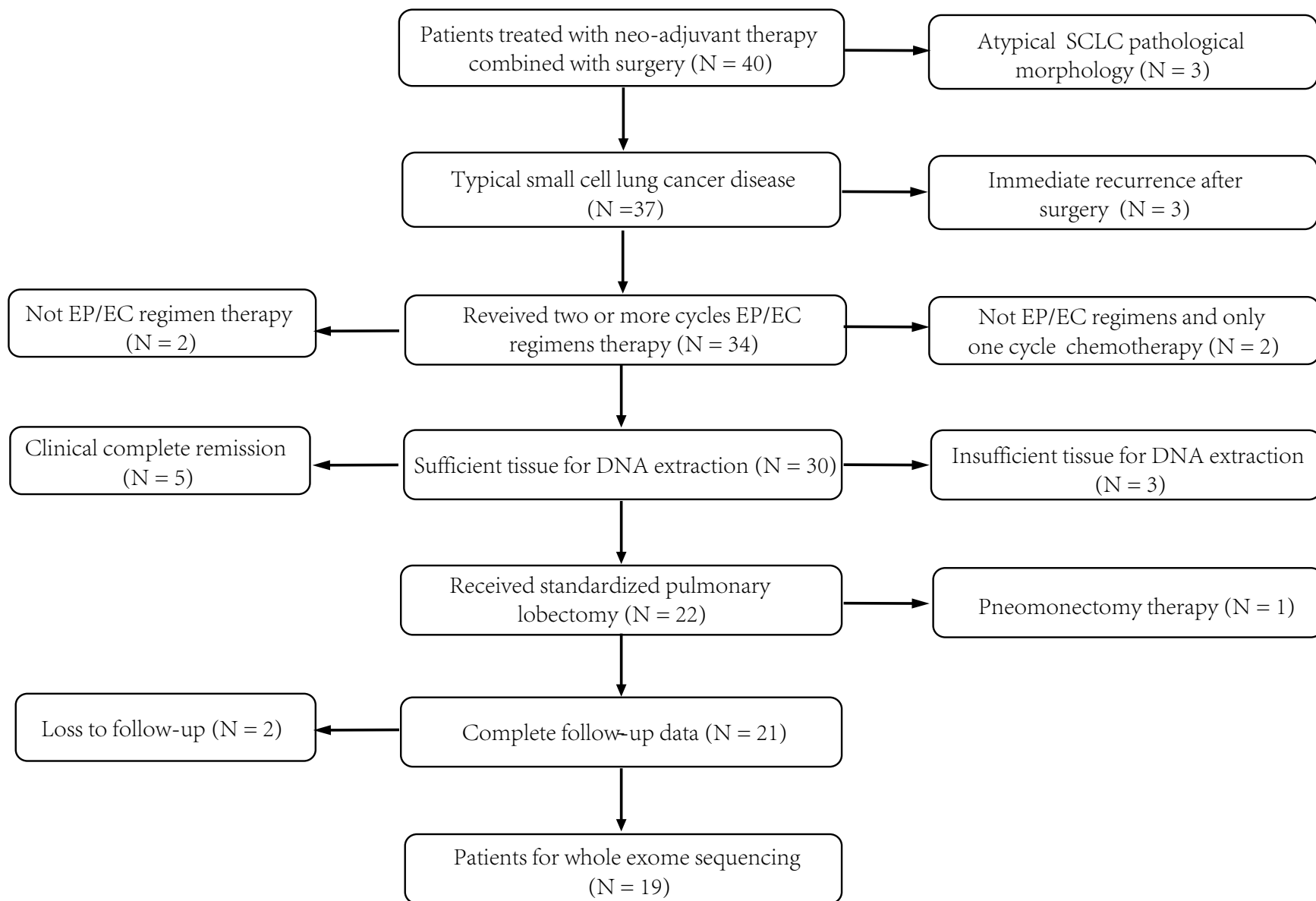

Supplement: Supplementary file 1 — Figure S1. [file CAM4-12-1035-s006.pdf]

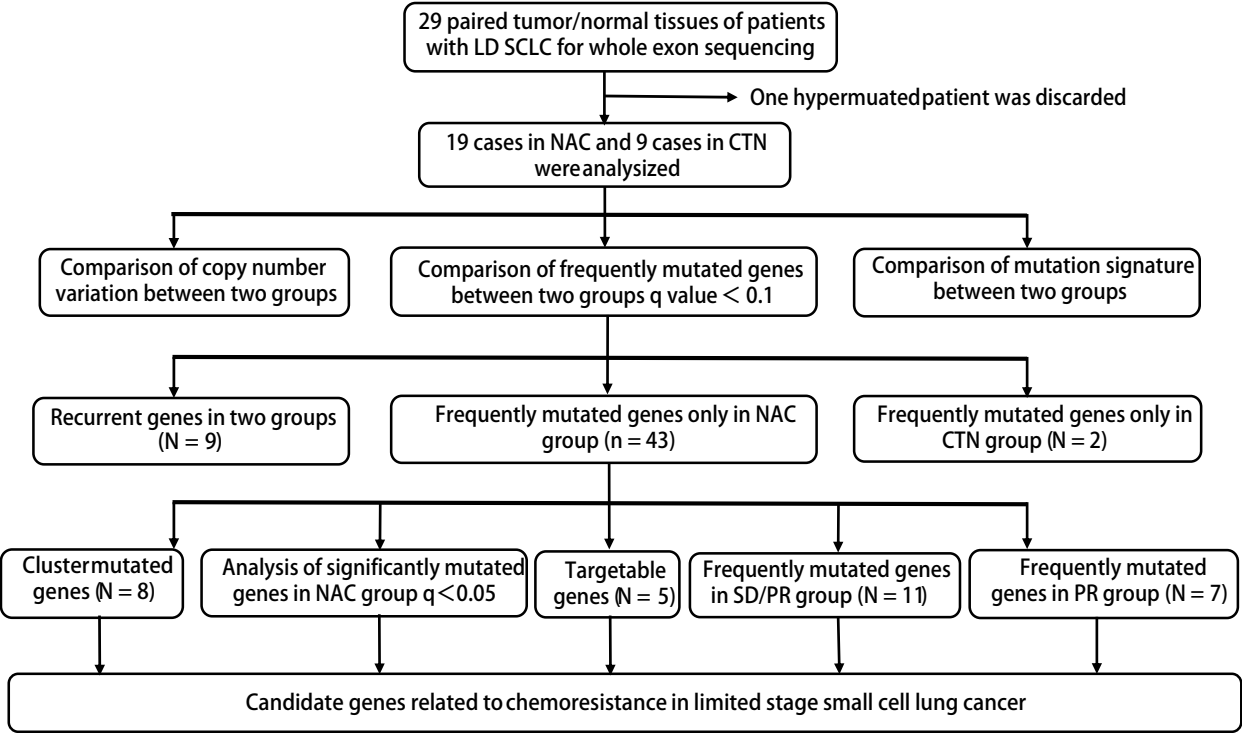

Supplement: Supplementary file 2 — Figure S2. [file CAM4-12-1035-s005.pdf]

a

COL11A1

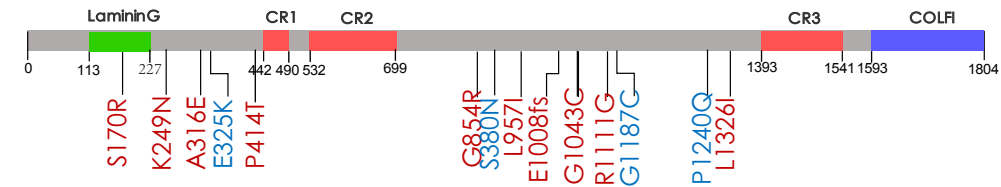

PKHD1

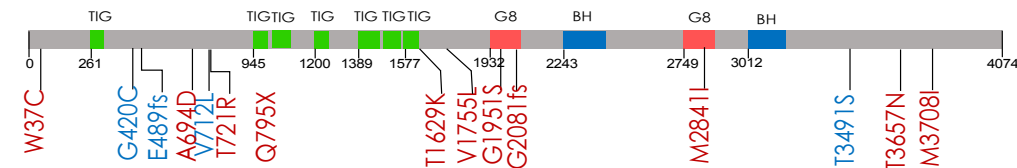

SYNE1

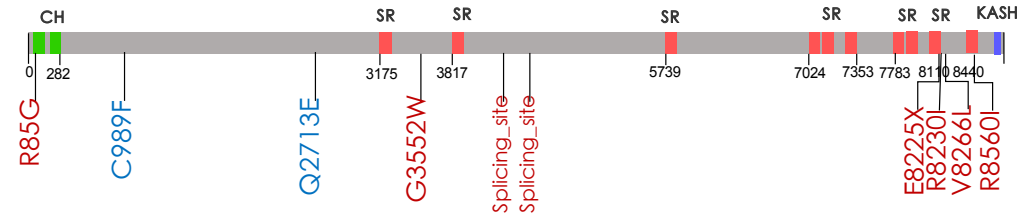

LRRC7

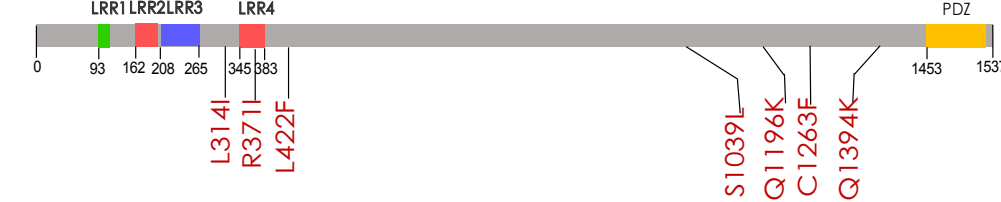

PCDH15

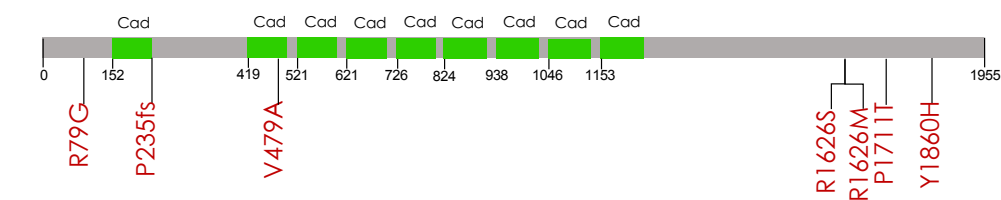

b

# Mutation Spectrum

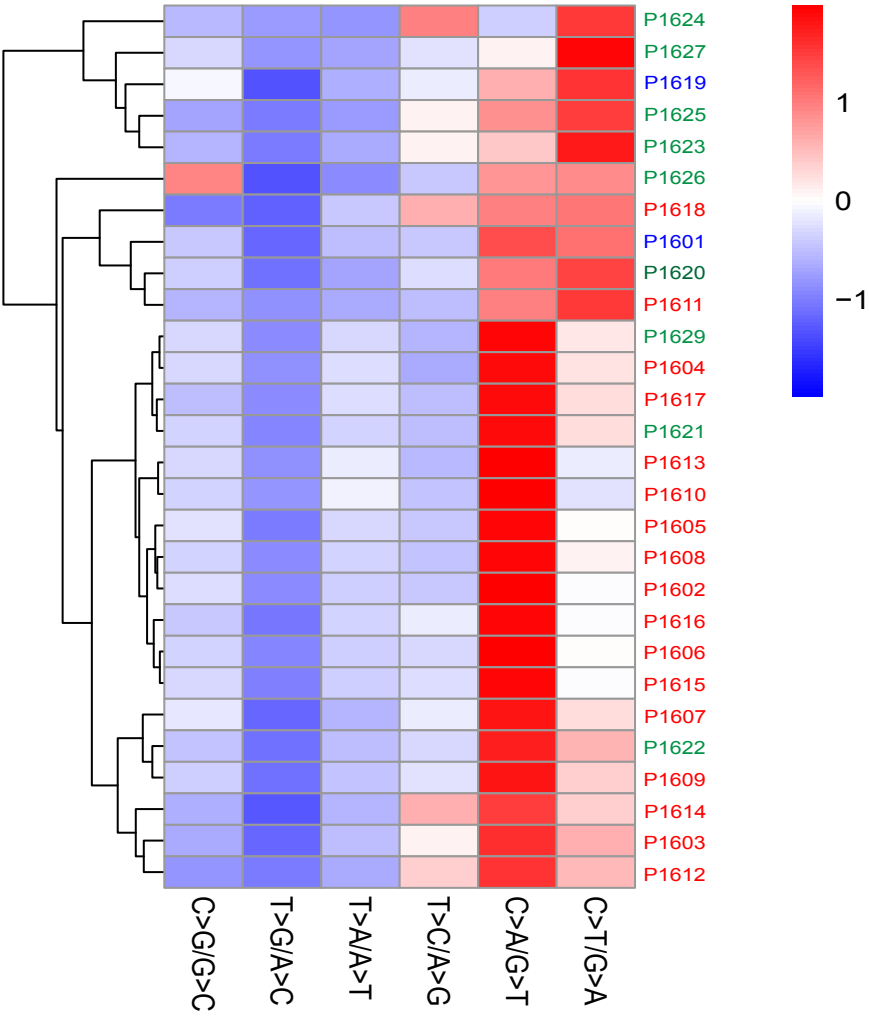

Supplement: Supplementary file 3 — Figure S3. [file CAM4-12-1035-s007.pdf]

a

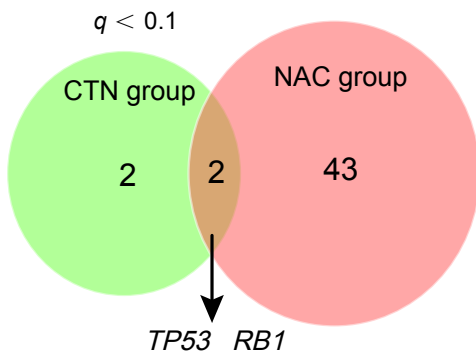

b

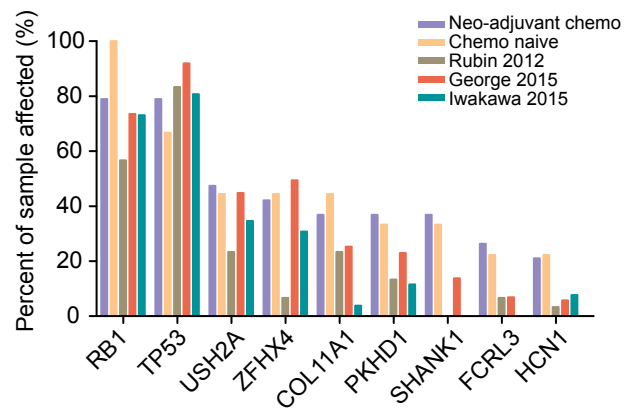

c

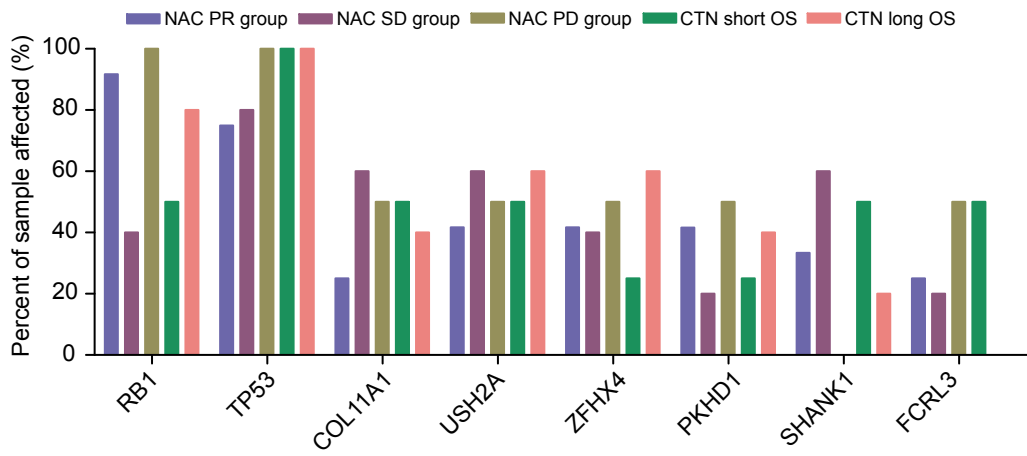

d

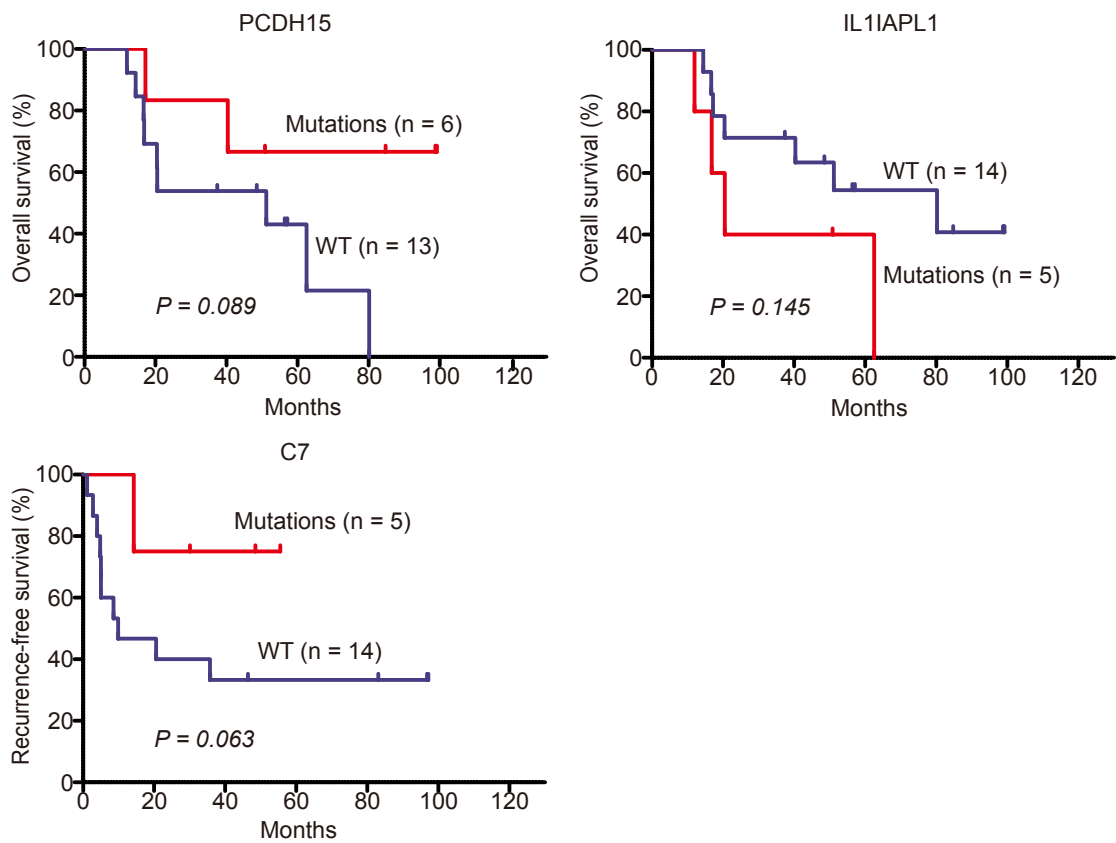

Supplement: Supplementary file 4 — Figure S4. [file CAM4-12-1035-s002.pdf]
