# Supplementary material for: Whole exome analysis reveals the genomic profiling related to chemo‐resistance in Chinese population with limited‐disease small cell lung cancer
Source: Cancer Med. 2022 Jun 23;12(2):1035–50. doi: 10.1002/cam4.4950 (PMC9883427; doi:10.1002/cam4.4950)
Supplement: Supplementary file 11 — Figure‐captions. [file CAM4-12-1035-s011.docx]

Figure S1. Schematic workflow for screening the patients with limited disease (LD) SCLC sequenced by WES.

Figure S2. Schematic flow for identifying candidate genes related to chemotherapy resistance in LD SCLC.

Figure S3. The frequently mutant genes and mutation signatures in NAC group are clustering existence. a. Representative schematics of recurrent genes like COLL11A1, PKHD1,SYNE1, LRRC7 and PCDH15 exhibit significant clustering of mutations in respective protein domains. Red fond and blue fond represent mutations in NAC group and CTN group, respectively. b. Clustered heatmap of mutation signatures. Color bar indicates the fraction of signatures. Samples are listed in right panel. Red, patients with efficacy of PR and SD; Blue, efficacy of PD; Green, CTN group.

Figure S4. Comparison of frequently mutated genes between NAC and CTN group. a. Venn diagram demonstrates the differences of frequently mutant genes between NAC and CTN group. b. Nine genes are frequently mutant in NAC group and other three studies with similar frequency. c. Eight genes are similarly frequent in every subgroup of NAC and CTN groups. d.Survival analysis of patients in NAC group based on mutant status of PCDH15, IL11APL1 and C7. Determined by log‐rank test.

Table S1. Basic characteristics of patients with limited‐stage small cell lung cancer treated with neo‐adjuvant chemotherapy combined with surgery (N = 40).

Table S2. Univariate and multivariate analyses for the RFS and OS of patients with SCLC treated with neo‐adjuvant chemotherapy and surgery.

Table S3. Quality control of somatic mutations in 28 patients with LD SCLC.

Table S4. The detailed information of somatic mutations in 28 patients with LD SCLC.

Table S5. Comparison of significantly mutated genes (q＜0.05) between neo‐adjuvant chemotherapy and chemotherapy naive group.

Table S6. Comparison of significantly mutated genes in different studies.
